# Increased Gaming During COVID-19 Predicts Physical Inactivity Among Youth in Norway—A Two-Wave Longitudinal Cohort Study

**DOI:** 10.3389/fpubh.2022.812932

**Published:** 2022-02-14

**Authors:** Ellen Haug, Silje Mæland, Stine Lehmann, Ragnhild Bjørknes, Lars Thore Fadnes, Gro Mjeldheim Sandal, Jens Christoffer Skogen

**Affiliations:** ^1^Department of Health Promotion and Development, Faculty of Psychology, The University of Bergen, Bergen, Norway; ^2^Department of Teacher Education, NLA University College, Bergen, Norway; ^3^Department of Global Public Health and Primary Care, Faculty of Medicine, University of Bergen, Bergen, Norway; ^4^Bergen Addiction Research, Department of Addiction Medicine, Haukeland University Hospital, Bergen, Norway; ^5^Department of Psychosocial Science, Faculty of Psychology, The University of Bergen, Bergen, Norway; ^6^Department of Health Promotion, Norwegian Institute of Public Health, Bergen, Norway; ^7^Alcohol and Drug Research Western Norway, Stavanger University Hospital, Stavanger, Norway; ^8^Centre for Evaluation of Public Health Measures, Norwegian Institute of Public Health, Oslo, Norway

**Keywords:** gaming, inactivity, COVID-19, youth, two-wave study

## Abstract

**Background:**

A concern for the COVID-19 measures and the potential long-term consequences the measures may have on physical inactivity and gaming among youth.

**Objectives:**

Examine the stability and change in internet and offline gaming and the association with physical inactivity among adolescents in Norway during the pandemic.

**Methods:**

A total of 2940 youth (58% girls) aged 12–19 years participated in an online longitudinal two-wave survey during the first Norwegian national lockdown in April 2020 (t1) and in December 2020 (t2). Gaming behavior and physical activity status were assessed at both time points. Age, gender, and socioeconomic status were included as covariates.

**Results:**

Among boys, 41% reported gaming a lot more and 35% a little more at t1 compared to before the national lockdown. The corresponding numbers for girls were 14 and 23%, respectively. In fully adjusted analysis, a pattern of increased gaming at t1 followed by an additional increase in gaming reported at t2 was associated with physical inactivity at t1 (OR = 2.10, *p* < 0.01) and t2 (OR = 2.45, *p* < 0.001). Participants gaming more at t1 followed by a reduction at t2 had higher odds of inactivity at t1 (OR = 1.88, *p* < 0.01). Youth reporting no gaming at t1 had lower odds for inactivity at this time point (OR = 0.67, *p* < 0.05).

**Conclusions:**

Increased gaming among many youths and a relationship with physical inactivity was observed during the first phase of the COVID-19 pandemic. To counteract the negative long-term impacts of COVID-19 restrictions, public health initiatives should emphasize the facilitation of physical activity in youth and develop effective strategies to prevent problematic gaming.

## Introduction

The COVID-19 outbreak has made a significant impact on young people's everyday life. From a public health perspective, children, and adolescents have so far been relatively protected from severe symptoms from the SARS-CoV-2 virus infection (1, 2). However, strong concerns are raised regarding the consequences of the intrusive disease-suppressive measures that have caused substantial changes in the daily lives of youth (3, 4). Emerging evidence indicates that these measures themselves may affect young people's mental health (5–7) and health behaviors (8, 9). One major worry relates to the short and long-term effects of increased physical inactivity and sedentary behaviors, such as watching TV, computer use, and gaming (1, 8, 10, 11). Screen-based activities are an integral part of young people's lives. Playing games on computers and other electronic devices as a leisure-time activity has become more and more popular (12). Moreover, in a period of social distancing measures and limited opportunities for meeting face-to-face and for organized activities, screen-based activities became increasingly important to young people, both for entertainment purposes and as a valuable arena to stay socially connected (13). However, this might come at the expense of physical activity. While physical activity among young people is well documented to affect wellbeing positively and contribute to preventing the onset of most chronic diseases, sedentary behaviors are associated with a heightened risk of obesity and cardiometabolic complications (14).

The balance between young peoples' screen-based activities and physical activity involvement was a public health concern before COVID-19 (15). According to the displacement hypothesis (16, 17), the amount of time youth devote to screen activities can displace the time they participate in physical activity (17). In a meta-analysis of primary studies published before the COVID-19 pandemic, the association between sedentary behaviors and physical activity in young people was negative but small, suggesting that these behaviors did not directly displace one another (18). Nevertheless, limited sports opportunities, restricted recreational facilities, and no travel time during school closure may have affected this relationship during the COVID-19 pandemic (19). Adding to this assumption is another perspective with particular relevance under the COVID-19 outbreak, the “Structured Days Hypothesis” (20), postulating that the presence of structure within regular school days increases daily physical activity and decreases sedentary screen-time. In contrast, the absence of “structure” could be one of the reasons why some children return to school, after longer holidays, with accelerated weight gain and a decrease in cardio-respiratory fitness (20).

During the COVID-19 pandemic, studies worldwide have indicated an overall increase in screen-time among young people (21–26), and video game playing has reached an all-time high (27). In March 2020, the global gaming industry launched the campaign #PlayApartTogether# to support public efforts in encouraging people to practice physical distancing in agreement with the World Health Organization (28). In most countries, the observed increase in screen-time has been accompanied by a decrease in physical activity (21–24). However, a German study found a rise in overall physical activity, despite a decrease in sports activity, due to an increase in habitual activities, such as walking and cycling (25). The authors conclude that the differential findings across countries may be related to contextual factors, such as policy actions, restrictions, and the rate of COVID-19 infections that directly affect behavior (25). In Norway, a nationwide lockdown was announced on March 12th, 2020, to suppress the outbreak (29). As a result, schools were closed and replaced with digital home-schooling. Organized sport and other leisure time activities were suspended, and young people could only have physical contact with the nearest family and 1-2 regular friends to play with outdoors. At the same time, the Norwegian Government strongly encouraged the public to spend time outdoors and be physically active while keeping a distance from others. The nationwide lockdown lasted for two months. Still, the period after has been characterized by repeated combinations of national, and local measures with unpredictability and infrequent opportunities for attending school, organized leisure activities, and meeting friends until the National reopening of the country on September 25th, 2021.

There are few studies exploring specific screen-based behaviors during the COVID-19 pandemic and the direct relationship with physical inactivity among youth. This two-wave longitudinal study aims to examine changes in gaming behavior and physical inactivity among youth aged 12–19 years during the COVID-19 induced lockdown in Norway in April 2020 and again in December 2020. The purpose is twofold. First, to examine the stability and change in internet and offline gaming behavior and the association with physical inactivity. Second, to examine if specific patterns of gaming behavior over time predicted physical inactivity. Age, gender, and socioeconomic status was controlled for.

## Materials and Methods

### Design, Procedure, and Participants

COVID-19 Young is a study of youth aged 12–19 within the municipality of Bergen. The study is part of a larger population-based study called the Bergen in Change (BiE-study). The study consists of two cohorts: Cohort 1 was youth aged 12–15 years whose parents had taken part in the BiE-study (30) and given their consent to us to invite their child to participate in the COVID-19 Young study. A total of 1,565 youth was contacted in Cohort 1. Cohort 2 was youth aged 16–19 years attending high schools in the municipality of Bergen. The County Municipality provided phone numbers from their pupil contact registers. A total of 5947 youth was contacted in Cohort 2.

The first wave of data collection lasted from the 27th of April, during the 7th week of lockdown, to the 11th of May (t1). The second wave of data was collected between the 16th of December 2020 and the 10th of January 2021 (t2). During t2, there were local restrictions with some schools partly continuing digital home-schooling to maintain social distancing measures, and most sports- and leisure activities were put on hold. The procedures were the same for cohorts 1 and 2 and on both time points. Eligible youth were invited via SMS with a link to a secure online platform containing an information letter and an online survey. The estimated time to complete the survey was 15 minutes. Two SMS reminders were given, and all participating youth were included in a lottery for a new cellphone.

A total of 7512 youth was invited to participate. Of these, 843 (54%) in cohort 1 and 2154 (36%) in cohort 2 responded, yielding a total of 2997 (40%) youths completing parts of, or the whole survey. The mean age was 16 years (*SD* 1.7), 59% female, and most participants reported living with both parents (77%), being born in Norway (93%), and living with siblings (71%). All participants from wave one were invited to answer the second survey, and a total of 1598 (53% of the sample at t1) responded.

### Measures

#### Sociodemographic Information

Demographic information included self-reported gender and age. Socioeconomic status (SES) was measured by self-rated family affluence collected at t2 by the question, “How well off do you think your family is compared to others?”. The response categories were “Better off”, “About the same” and “Worse off”.

#### Gaming Behavior

To examine gaming behavior, the following explanatory text was given. “*By playing/gaming, we mean all internet and offline gaming, on the phone, PC, tablet, console. We do not count in board games, gambling or internet use in school or work contexts or pure social media*”. To assess how much youth played during the initial lockdown in Spring 2020, the following question was asked: “After the school closed, how much have you played/gamed”? To assess gaming behavior in the period after the school was closed during the lockdown, the following question was asked at t2; “*After the summer holidays, how much have you played/gamed compared to when the school was closed this spring?*”. The answer categories for both time points were: “A lot more”; “a little more”; “about the same”; “a little less,” “a lot less,” and “no gaming.” A variable assessing the joint gaming change over both time points was constructed and had five levels (1=more and increasing (i.e. reporting increase at both time points), 2=more and decreasing (i.e., reporting more gaming at t1 but less at t2), 3=increasing (i.e., “about the same” at t1 but more gaming at t2), 4=about the same (i.e. those reporting “about the same” at both time points), and 5=less (i.e. those reporting less gaming at t1 and less or ‘about the same' at t2) 5=no gaming (i.e. those reporting no gaming at both time-points).

#### Physical Activity Status

Physical activity status was measured using one item from the physical wellbeing dimension of the KIDSCREEN-27 Quality of Life Questionnaire (31). With reference to: “*When you think about the last week*”, the respondents were asked to answer the question: **“**Have you been physically active (e. g. running, climbing, biking)?” on a five-point Likert scale (1=not at all, 2=slightly, 3=moderately, 4=very, and 5=always or extremely). For the purpose of this study, the answers were dichotomized into active (3–5) and inactive (1, 2).

### Statistics

First, summary statistics of the included study variables across gender were estimated and presented in Table 1. For each gender, the distribution of the study variables was expressed as proportions, and potential gender differences were assessed using χ^2^-statistics. Next, the associations between reported change in gaming and physical activity status, at t1 and t2, respectively, were estimated using logistic regression models (Table 2). Using the self-reported change in gaming at t1 as an independent variable and physical activity status at each time point as dependent variables, three regression models were computed; a crude model, a model adjusted for age and gender, and a model adjusted for age, gender, and socioeconomic status (at t2). Thereafter, the association between change in gaming behavior across the two time points and physical inactivity, at t1 and t2 respectively, were estimated using logistic regression models (Table 3). Using the joint categorization of gaming change over the two time points as an independent variable, and physical activity status at each time point as dependent variables, three regression models were computed for each time point; a crude model, a model adjusted for age and gender, and a model adjusted for age, gender and socioeconomic status (at t2). Lastly, the potential moderating effect of gender on the association between gaming status and physical activity status was investigated in all regression models using likelihood ratio tests comparing base models with nested models. To keep as much information as possible, pairwise deletion was employed in the regression models.

**Table 1:** Summary of study variables across gender.

	***N***	**Boys (%)**	**Girls (%)**	***P***
		**(*N* = 1,246)**	**(*N* =1,694)**	
Age, t1				*p* = 0.123
12	161	6	5	
13	201	8	6	
14	187	6	7	
15	213	8	7	
16	688	22	25	
17	1,097	37	37	
18+	389	13	14	
Socioeconomic status, compared to others at t2				***p*** < **0.001**
Better off	349	30	19	
About the same	1043	64	71	
Worse off	133	6	10	
Gaming, t1				***p*** < **0.001**
A lot more	686	41	14	
A little more	762	35	23	
About the same	501	14	21	
A little less	84	4	3	
A lot less	32	1	1	
No gaming	652	4	38	
Gaming, t2				***p*** < **0.001**
A lot more	172	20	9	
A little more	255	22	18	
About the same	348	30	25	
A little less	141	15	8	
A lot less	65	9	3	
No gaming	306	4	36	
Physical activity status, t1				***p*** **=** **0.010**
Active	1718	69	64	
Inactive	872	31	36	
Physical activity status, t2				***p*** **=** **0.011**
Active	615	60	52	
Inactive	498	40	48	
Categorization of gaming change over two time points				***p*** < **0.001**
More and increasing	298	37	17	
More and decreasing	377	43	23	
Increasing	106	5	11	
About the same	133	6	14	
Less	126	7	12	
No gaming	192	2	24	

*Bold indicates significant differences between groups, based on χ^2^-statistics*.

**Table 2:** Associations between reported change in gaming and being physical inactive at first assessment (t1) and second assessment (t2) estimated by logistic regressions.

	**Inactive T1**			**Inactive T2**		
**Gaming frequency**	**Crude** **(*N* = 2,608)**	**Adjusted for age and gender** **(*N* = 2,576)**	**Adjusted for age, gender and SES at t2** **(*N* = 1,379)**	**Crude** **(*N* = 1,084)**	**Adjusted for age and gender** **(*N* = 1,071)**	**Adjusted for age, gender and SES at t2** **(*N* = 1,070)**
A lot more	1.42^**^ **(1.11–1.82)**	1.74^***^ **(1.34–2.26)**	1.82^**^ **(1.26–2.63)**	1.50^*^ **(1.03–2.16)**	1.88^**^ **(1.26–2.18)**	1.87^**^ **(1.25–2.80)**
A little more	1.09 (0.85–1.39)	1.23 (0.96–1.59)	1.34 (0.94–1.91)	1.19 (0.83–1.69)	1.49^*^ **(1.02–2.18)**	1.51^*^ **(1.03–2.20)**
About the same	***Ref***.	***Ref***.	***Ref***.	***Ref***.	***Ref***.	***Ref***.
A little less	0.87 (0.52–1.45)	1.01 (0.60–1.71)	0.62 (0.27–1.43)	0.88 (0.40–1.96)	0.88 (0.38–2.05)	0.91 (0.39–2.13)
A lot less	0.67 (0.28–1.60)	0.76 (0.31–1.84)	0.96 (0.24–3.81)	0.86 (0.20–3.71)	0.98 (0.22–4.46)	0.98 (0.21–4.57)
No gaming	0.83 (0.64–1.08)	0.68^**^ **(0.52–0.89)**	0.67^*^ **(0.46–0.98)**	1.00 (0.69–1.46)	0.82 (0.56–1.22)	0.85 (0.57–1.27)

*Estimates in bold =*

*** *p < 0.001*,

** *p < 0.01*,

* *p < 0.05*.

*Odds ratios (OR) are presented with 95% confidence intervals. Ref., reference category*.

**Table 3:** Association between categorization of change in gaming and being inactive at first assessment (t1) and second assessment (t2) estimated by logistic regressions.

	**Inactive T1**			**Inactive T2**		
**Gaming change over two time points**	**Crude** ***N* = 1,202)**	**Adjusted for age and gender** **(*N* = 1,186)**	**Adjusted for age, gender and SES at t2** **(*N* = 1,185)**	**Crude** **(*N* = 1,082)**	**Adjusted for age and gender** **(*N* = 1,069)**	**Adjusted for age, gender and SES at t2** **(*N* = 1,068)**
More and increasing	1.80^*^ **(1.14–2.84)**	2.10^**^ **(1.30–3.38)**	2.08^**^ **(1.29–3.37)**	1.80^**^ **(1.16–2.78)**	2.48^***^ **(1.55–3.97)**	2.45^***^ **(1.53–3.94)**
More and decreasing	1.67^*^ **(1.07–2.61)**	1.85^**^ **(1.16–2.94)**	1.88^**^ **(1.18–2.99)**	1.20 (0.79–1.83)	1.51 (0.96–2.38)	1.52 (0.96–2.39)
Increasing	1.23 (0.69–2.18)	1.21 (0.67–2.18)	1.22 (0.68–2.20)	1.19 (0.68–2.07)	1.19 (0.67–2.13)	1.16 (0.65–2.09)
About the same	***Ref***.	***Ref***.	***Ref***.	***Ref***.	***Ref***.	***Ref***.
Less	1.13 (0.65–1.97)	1.17 (0.66–2.06)	1.20 (0.68–2.12)	0.87 (0.51–1.47)	0.92 (0.53–1.59)	0.94 (0.54–1.63)
No gaming	0.87 (0.52–1.46)	0.70 (0.41–1.20)	0.73 (0.43–1.25)	1.25 (0.78–2.00)	1.04 (0.64–1.71)	1.09 (0.66–1.78)

*Estimates in bold =*

*** *p < 0.001*,

** *p < 0.01*,

* *p < 0.05*.

*Odds ratios (OR) are presented with 95% confidence intervals. Ref., reference category*.

## Results

### Descriptives

A total of 2,940 participants were eligible for analyses in the present study. There were no gender differences with respect to age at t1, but boys reported a higher socioeconomic status at t2 than girls (Table 1). For gaming at both t1 and t2, a higher proportion of the boys reported more gaming compared to the girls. Furthermore, a substantially higher proportion of girls reported “no gaming” (38 vs. 4% at t1, and 36 vs. 4% at t2). With respect to physical inactivity status, a higher proportion of girls reported being inactive at both time points compared to boys. The joint categorization of gaming change over two time points indicated that boys were more likely to a report change in gaming across time points, including “more and increasing” and “more and decreasing,” compared to girls. Also, for this variable, there were substantial gender differences in no gaming (24% of the girls vs. 2% of the boys).

### Association Between Gaming Behavior and Physical Activity Status

Reporting a lot more gaming at t1 was associated with a higher odds of physical inactivity at both t1 and t2 (Table 2). At both time points, the strength of the association increased markedly after adjustment for age and gender, while further adjustment for socioeconomic status only changed the estimates marginally. Reporting a little more gaming at t1 was also associated with a higher odds of physical inactivity at t2, but only in adjusted models. Furthermore, “no gaming” was associated with a lower odds of physical inactivity at t1 in the adjusted models. Results from likelihood ratio tests did not indicate the presence of a moderating effect of gender on the association between gaming status and physical activity status [gaming at t1 and physical activity status at t1 (*p* = 0.245) and physical activity status at t2 (*p* = 0.992)].

For the joint measure of gaming change across both time points, “more and increasing” gaming was associated with a higher odds for physical inactivity at both t1 and t2 (Table 3). At both time points, the strength of the association increased after adjustment for age and gender, especially in relation to physical inactivity at t2, while socioeconomic status had little impact on the estimates. For “more and decreasing” gaming a significant association was only observed for physical inactivity at t1, and not at t2. Again, the strength of the association became more pronounced in the age- and gender-adjusted models compared to the crude model, and socioeconomic status did not alter the estimated strength to any extent. Results from likelihood ratio tests did not indicate the presence of a moderating effect of gender on the association between the joint measure of gaming change and physical activity status [physical activity status at t1 (*p* = 0.779) and physical activity status at t2 (*p* = 0.679)].

## Discussion

The results of this two-wave longitudinal study among youth aged 12-19 years in Norway reveal that increased gaming was common during the initial lockdown in April 2020 (t1). Among boys, 41% reported a lot more and 35% a little more gaming, whereas the numbers for girls were 14 and 23%, respectively. Participants that reported a lot more gaming at t1 compared to before the lockdown had higher odds of being physically inactive at this time point and nine months later in December 2020 (t2). Participants that reported a little more gaming had only increased odds of being inactive at t2. In comparison, youth that was not gaming at t1 had lower odds of inactivity at this time point. Furthermore, a gaming pattern characterized by more gaming at t1 followed by a further increase reported at t2, was associated with being inactive at both time points. A pattern of an initial increase at t1 followed by a decrease at t2 was only associated with being inactive at t1.

Increased gaming in parts of the youth population during the times of the COVID-19 pandemic causes public health concerns, as gaming has been associated with physical inactivity, poor mental health, and sleep problems (32). Sedentary behaviors and physical activity are relatively stable behaviors that are traceable from adolescence to later in life (33, 34). Therefore worries have been raised that the disease-suppressive measures taken under the COVID-19 pandemic will add to the already ongoing trend of physical inactivity among youth, with great health impacts (1). Thus, to counteract the potential adverse effects of inactivity and gaming after the first phase of the COVID-19 pandemic and similar public health crises in the future, public health initiatives should contain information on effective parental strategies to prevent problematic video gaming (35) in the post-pandemic aera. Furthermore, governments at national and local levels have an important window to optimize the opportunities for youth to engage in unorganized and organized physical activities to reverse physical inactivity linked to increased gaming during the pandemic.

In our study, a further increase in gaming after the schools re-opened in the fall semester of 2020 was almost as common as a decrease. This indication of an escalation of gaming among a considerable group of youth is a concern as it might reflect impaired control typically seen with a gaming disorder (36). In the 11^th^ Revision of the International Classification of Diseases (ICD-11), gaming disorder was included and defined as a pattern of gaming behavior (“digital-gaming” or “video-gaming”) characterized by “*impaired control over gaming, increasing priority given to gaming over other activities to the extent that gaming takes precedence over other interests and daily activities, and continuation or escalation of gaming despite the occurrence of negative consequences”* (36). The inclusion of the gaming disorder has been debated, largely based on a lack of consensus on the diagnostic criteria (37). Nevertheless, it has been argued that the possibility of young people developing gaming disorder in the time of COVID-19 could be higher due to the valid reason for them to engage in screen-based activities (38). Our results highlight youth's possible risk of increased gaming over health-related behavior, specifically physical activity. The overall increase in gaming among a substantial proportion of those that initially increase their gaming in the first face of the pandemic is a reason for increased awareness in the aftermath of the COVID-19 period. Nevertheless, young people's gaming practices may also have had beneficial effects in retaining a social life and providing a legitimate social arena for maintaining friendships and coping with boredom in the dramatic and sudden shift from everyday routines to days regulated by measures (13).

The majority of those that initially increased their gaming during the lockdown reported a decrease in the fall semester of 2020 when the schools re-opened. This group did not have increased odds of being inactive at the second time point. Thus, our findings support that the gaming behaviors partly seem to be related to the specific contextual situation during the lockdown, in line with the “Structured Days Hypothesis” (20). The increased gaming reported during the lockdown could be induced by the measures to repress the spreading of the virus. A recent study documented that the lockdown led to major interferences in everyday life with more unstructured days for many youths (3). Less gaming among many participants in the fall of 2020 after the lockdown could reflect a return to a more normal life, such as physical attendance in school and a return to some of the organized leisure time activities. This may have resulted in less need or time for gaming. Future research may inform us further on whether increased gaming activity during COVID-19 will lead to a heightened prevalence of problematic gaming behaviors among youth or if this increase is more representative of a context-based adaptive behavior in absence of structure, and maintains social needs in a period of lock-down.

Our results add to the emerging literature that has found an increase in screen time behaviors and a decrease in physical activity among youth in the first phase of the COVID-19 outbreak (21–25) and demonstrate a positive relationship between increased gaming and physical inactivity over an extended period under the pandemic. The findings align with a potential replacement of physical activity among youth reporting increased gaming during the pandemic, thus supporting the displacement hypothesis (17). However, the findings could also be explained by other factors. A higher proportion of youth was inactive at the second time point in the winter, suggesting an overall increase in sedentary activities. There were still various local restrictions during fall 2020 and unavailability of activities, especially indoor sporting, that the participants might have engaged in under normal circumstances. Particularly those attending upper secondary school had to partly continue with digital home-schooling for the schools' institutions to fulfill the required social distancing measures. This could have contributed to fewer youths returning to their organized activities and may also have resulted in some dropping out of sports. There is currently limited evidence on the impact of COVID-19 on youth sport. Theoretical assumptions of a “generation lost” to sports have been put forward (39), and challenges for sports to attract volunteers and participants back into sports have been reported (40). Wintertime is also viewed as less favorable for outdoor activities (e.g., fewer hours of daylight, colder, more wind, and more rain) (41). A pre-pandemic national survey of 15-year old Norwegians did find higher odds for meeting objectively assessed physical activity recommendations in spring compared to winter, but no association between mean physical activity and season (42). Under the local disease suppressive measure affecting organized activities, the effect of the season might have been greater.

There were substantial gender differences, with a higher number of boys reporting more gaming than girls. Furthermore, one of three girls did not game at all, while a few boys reported no gaming. Similar and substantial gender-related differences in gaming have typically been observed in studies worldwide (43). Girls might have involved themselves more in other screen-based activities in this period. For instance, girls are more likely than boys to be intensive users of electronic media communication (44) and have reported more social media use during the COVID-19 lockdown than boys (45). In the current study, more girls than boys reported being inactive at both time points, suggesting a general tendency of a higher preference for sedentary pursuits. However, our results showed no moderating effect of gender in the association between gaming and inactivity, implying that this relationship did not differ across gender. It should also be noted that at both time points, the strength of the associations between gaming and inactivity increased substantially after adjustment for age and gender. This demonstrates a negative confounding effect of these variables, mostly driven by adjustment for gender (data not shown).

### Strengths and Limitations

A strength of this study is the large sample of young people and its longitudinal design during the pandemic. Still, there was no comparison condition and no pre-pandemic assessment. Because of this, it is not possible to ascribe any changes to the impact of COVID-19. Further, the data were self-reported and thus prone to recall bias and social desirability (46). Both physical inactivity and gaming were assessed with single items. However, a recent review on subjective measures of sedentary behaviors did not find substantial differences between the criterion validity of a 1-item versus multiple-item questionnaires and concluded that the appropriate measure will depend on the nature of the study (47). Further, in a recent review of subjective measures assessing physical activity among children and youth, a single-item activity measure was the most reliable test-retest questionnaire in adolescents (48). We did not have the information on how the local measures to maintain social distancing affected the individual participant with regards to opportunities for physical school attendance and participation in organized sports activities; these factors were therefore not controlled for. Also, the characteristics of the parents for the youngest cohort suggest that this was a selected group of youth, whose parents had higher income and educational level compared to the non-consenting parents. Together with relatively low response rates, interpretations warrant caution as the findings might not be generalizable to the population as a whole. Even so, considering the generalizability, all schools were closed in April 2020 and many schools were partly closed in December in the geographic area of our sample. This is representative of the situation for many youths in Europe during the pandemic.

## Conclusion and Implications

The results demonstrate an increase in gaming in a substantial number of participants, particularly among boys, which was associated with increased odds for physical inactivity. This adds to the knowledge base on the impact of restrictions to suppress the COVID-19 outbreak among youth. To counteract the potential negative effects of inactivity and gaming due to the COVID-19 pandemic, public health initiatives should include a broad range of approaches to promote physical activity and develop effective strategies to prevent problematic gaming among youth.

## Data Availability Statement

The datasets presented in this article are not readily available because of national regulations on health research. It can be available from the corresponding author on reasonable request. Requests to access the datasets should be directed to ellen.haug@uib.no.

## Ethics Statement

The studies involving human participants were reviewed and approved by the Regional Committee for Medical and Health Research Ethics, Western Norway (project number 131560). Written informed consent to participate in this study was provided by the participants' legal guardian/next of kin.

## Author Contributions

EH, SL, RB, and SM have made substantial contributions to the conception and design of the study and the acquisition of data. JS analyzed the data and made substantial contributions to the interpretation. EH has drafted the manuscript. SM, SL, RB, JS, LF, and GS made critical revisions and provided intellectual content to it. All authors approved the final manuscript to be published.

## Funding

This project was co-founded by the University of Bergen and Bergen municipality. There was no external funding.

The author(s) thank the youth participating in this study for their time and effort. We also thank Guri Rørtveit, Benedicte Carlsen, Kjell Wolff, Trond Egil Hansen, and Øystein Hetlevik for their contribution in laying grounds for the study.

## Conflict of Interest

The authors declare that the research was conducted in the absence of any commercial or financial relationships that could be construed as a potential conflict of interest.

## Publisher's Note

All claims expressed in this article are solely those of the authors and do not necessarily represent those of their affiliated organizations, or those of the publisher, the editors and the reviewers. Any product that may be evaluated in this article, or claim that may be made by its manufacturer, is not guaranteed or endorsed by the publisher.
